# Association of *OGG1* and *MTHFR* polymorphisms with age-related cataract: A systematic review and meta-analysis

**DOI:** 10.1371/journal.pone.0172092

**Published:** 2017-03-02

**Authors:** Xiaohang Wu, Weiyi Lai, Haotian Lin, Yizhi Liu

**Affiliations:** State Key Laboratory of Ophthalmology, Zhongshan Ophthalmic Center, Sun Yat-sen University, Guangzhou, Guangdong, People’s Republic of China; Soochow University Medical College, CHINA

## Abstract

**Purpose:**

To discern and confirm genetic biomarkers that help identify populations at high risk for age-related cataract (ARC).

**Methods:**

A literature search was performed in the PubMed, Web of Science and China National Knowledge Internet databases for genetic association studies published before June 26, 2016 regarding ARC susceptibility. All genetic polymorphisms reported were systematically reviewed, followed by extraction of candidate genes/loci with sufficient genotype data in ≥3 studies for the meta-analysis. A random/fixed-effects model was used to calculate the pooled odds ratios and 95% confidence intervals to evaluate the associations considering multiple genetic models. Sensitivity analysis was also performed.

**Results:**

A total of 144 polymorphisms in 36 genes were reported in the 61 previous genetic association studies. Thereby, three polymorphisms of two genes (8-oxoguanine DNA glycosylase-1 [*OGG1*]; methylenetetrahydrofolate reductase NADPH [*MTHFR*]) in eight studies were included in the meta-analysis. Regarding the *OGG1*-rs1052133, the *GG* (OR = 1.925; 95%CI, 1.181–3.136; *p* = 0.009) and *CG* (OR = 1.384; 95%CI, 1.171–1.636; *p*<0.001) genotypes indicated higher risk of ARC. For the *MTHFR* gene, the *CC+TT* genotype of rs1801133 might be protective (OR, 0.838; 95%CI, 0.710–0.989; *p* = 0.036), whereas the *AA+CC* genotype of rs1801131 indicated increased risk for the mixed subtype (OR = 1.517; 95%CI, 1.113–2.067; *p* = 0.008).

**Conclusions:**

Polymorphisms of *OGG1* and *MTHFR* genes are associated with ARC susceptibility and may help identify populations at high risk for ARC.

## Introduction

Age-related cataract (ARC), also known as senile cataract, remains the leading cause of blindness worldwide, accounting for approximately 80% of senile blindness cases[1]. ARC is the gradual opacification of the aging lens, which hinders light transmission from outside the eyeball to the retina[2]. Multiple interactive factors have been demonstrated to participate in the complex cataractogenesis process, among which genetic background is drawing increasing attention and is accepted as the most principal causative factor, constituting half the risk [3, 4].

Genetic polymorphisms have been recognized as partly contributing to the genetic risk factors for cataract and increasing efforts are focused on identifying the associations between genetic polymorphisms and cataract susceptibility [5–7]. The polymorphisms of genes encoding antioxidant enzymes, such as glutathione S transferase (*GST*)[5, 8, 9], genes encoding DNA repair enzymes, such as xeroderma pigmentosum complementation group D (*XPD*) and X-ray cross-complementing group 1 (*XRCC1*)[7, 10, 11] have been confirmed as associated with ARC susceptibility.

However, the ARC genetic association profile has not been reviewed to date. Additionally, the polymorphism susceptibility and the number of individual genes vary across different studies. We therefore undertook a review and meta-analysis to provide a general assessment of existing original studies in this field and to discern or confirm new genetic biomarkers that may facilitate the identification of population at high risk for ARC.

## Materials and methods

### Literature search

A literature search was performed by two reviewers (Xiaohang Wu and Weiyi Lai) in the PubMed, Web of Science and China National Knowledge Internet (CNKI) electronic databases for genetic association studies concerning ARC susceptibility published before June 26, 2016. All the genetic association studies with ARC identified were systematically reviewed followed by extraction of candidate genes/loci with sufficient genotype data in ≥3 studies for the meta-analysis. We also manually assessed the reference lists of all the retrieved original studies, review articles and conference abstracts using the electronic databases listed above. In our literature search, combinations of items were used including cataract, polymorphism, and the full name/abbreviation of the candidate genes. The detailed search strategy in PubMed was listed as follows:

("polymorphism, genetic"[MeSH Terms] OR ("polymorphism"[All Fields] AND "genetic"[All Fields]) OR "genetic polymorphism"[All Fields] OR "polymorphism"[All Fields]) AND ("cataract"[MeSH Terms] OR "cataract"[All Fields]).

For more details, please refer to S1 Appendix in the supplementary information.

### Eligibility criteria

We considered studies eligible for the meta-analysis if they fulfilled the following criteria: (1) original case-control and cohort studies that evaluated genetic association with age-related cataract susceptibility; (2) samples that consisted of unrelated individuals recruited from well-defined populations; (3) genotype and allele data of both case and control groups provided or calculable from the reported data; and (4) no identified previous systematic review or meta-analysis concerning the polymorphism locus, or the previous meta-analysis needed to be updated.

We excluded studies for the following reasons: (1) animal studies, case reports, reviews, duplicate publications or conference abstracts; (2) diagnosis of cataract not based on objective examination or medical records; and (3) studies published in languages other than English or Chinese.

### Study selection, data collection and risk of bias assessment

Two reviewers (Xiaohang Wu and Weiyi Lai) screened all the records independently. All disagreements were resolved through discussions with a third reviewer (Haotian Lin). After identifying all the eligible articles, two authors (Xiaohang Wu and Weiyi Lai) extracted the data and compared the results. We did not contact the authors of the eligible articles for additional data. A standardized data extraction form was used that included the first author, year of publication, population ethnicity, population characteristics (mean age and sex ratio, using the control group as the reference), definitions of case and control groups, sample sizes, involved genes and polymorphisms, allelic and genotypic counts of the case and control groups, and the genotype frequencies of different cataract subtypes when provided, for the purpose of stratified analysis. When the allelic counts were not reported, we calculated them using the genotype data. The results of Hardy-Weinberg equilibrium (HWE) test were also extracted from the control group using the chi-square test.

The quality of the included studies was assessed by two reviewers (Xiaohang Wu and Weiyi Lai) according to the Newcastle-Ottawa Scale (NOS) (available at http://www.ohri.ca/programs/clinical_epidemiology/oxford.asp). This scale uses a domain-based system to assess the quality of a study involving selection, comparability and exposure, with scores ranging from 0 (worst) to 9 (best).

### Data analysis

Meta-analysis was conducted for each of the candidate polymorphisms using a model-free approach [12]. No prior assumptions regarding genetic models were made. In brief, values of λ = 0, 0.5, and 1 indicate recessive, codominant and dominant models respectively. With λ higher than 1 or lower than 0, the overdominant model is favored. Other genetic models were also analyzed to aid the comprehensive assessment of the estimated association. The genetic models we used included allelic (*a* vs *A*), dominant (*aa+Aa* vs *AA*), recessive (*aa* vs *Aa+AA*), codominant (*aa* vs *AA* and *Aa* vs *AA*) and overdominant (*aa+AA* vs *Aa*) models, where ‘a’ and ‘A’ represent the mutant allele and the wild-type allele, respectively.

The pooled odds ratios (OR) and 95% confidence intervals (95% CI) were calculated for each polymorphism involving multiple genetic models with a fixed-effects model (the Mantel-Haenszel method) or random-effects model (the DerSimonian-Laird method) according to the interstudy heterogeneity. Heterogeneity among the included studies was assessed by the I^2^-based Q statistic test[13]. I^2^ values of 50% or more were considered to indicate substantial heterogeneity, and the random-effects model was then used; otherwise, the fixed-effects model was used. The significance of the pooled OR was determined by Z-test, with p<0.05 considered statistically significant. The Egger’s test was used to assess the publication bias with p<0.05 considered statistically significant. We also conducted sensitivity analysis to test the robustness of associations by sequentially omitting each of the included studies one at a time. All the data analysis was performed using the software STATA 13.0 (Stata Corporation, College Station, TX, USA).

## Results

### Review of the genetic polymorphisms reported in association studies with ARC

Our general literature search of all genetic association studies regarding age-related cataract generated a total of 61 selected studies, involving 144 polymorphisms in 36 genes. All these involved polymorphisms and references are listed in Table 1.

**Table 1:** Systematic review of the genetic polymorphisms in previous association studies regarding age-related cataract.

Gene	Full name	Role	Polymorphism	No. of related studies	Previous systemic review
***GST***	glutathione S transferase	Antioxidant enzyme	GSTM1, GSTM3, GSTT1, GSTP1, GSTO1, GSTO2	13[14–26]	Yes[5, 8, 9]
***NAT2***	N-acetyltransferase type 2	Antioxidant enzyme	NAT2*5A, NAT2*6A, NAT2*7A/B, NAT2*14A	2[27, 28]	No
***SOD***	Superoxide dismutase	Antioxidant enzyme	SOD1: rs17881180, rs2234694, rs17880135, rs2070424; SOD2: rs6917589, rs2842980, rs7855, rs5746151, rs5746136, rs4880, rs2758352; SOD3: rs2536512, rs1799895	2[29, 30]	No
***CAT***	Catalase	Antioxidant enzyme	rs7943316	1[29]	No
***GPX1***	Glutathione peroxidase	Antioxidant enzyme	rs1050450	1[29]	No
***XPD***	Xeroderma pigmentosum complementation group D	DNA repair enzyme (nucleotide excision repair pathway)	Codon 751, codon 312 (rs1799793)	5[31–35]	Yes[7, 10, 11]
***XRCC1***	X-ray cross-complementing group 1	DNA repair enzyme (base excision repair pathway)	Codon 399 (rs25487)	5[31–34, 36]	Yes[7, 10, 11]
***WRN***	Werner helicase	DNA repair enzyme (double-strand end resection pathway)	rs1346044, rs1801195, rs2230009, rs3087414, rs4733220, rs2725361, rs2725338, rs2725383, rs1863280, rs11574311	3[37–39]	No^a^
***APE1***	AP endonuclease-1	DNA repair enzyme (base excision repair pathway)	Codon 148, rs1760944	2[34, 36]	No
***ERCC6***	ERCC excision repair 6, chromatin remodeling factor	DNA repair enzyme (nucleotide excision repair pathway)	rs4838519, rs4253038	1[39]	No
***BLM***	Bloom syndrome RecQ like helicase	DNA repair enzyme (double-strand end resection pathway)	rs1063147, rs7183308, rs17273206, rs8027126, rs7175811, rs3815003, rs6496724	1[39]	No
***OGG1***	8-oxoguanine glycosylase-1	DNA repair enzyme (base excision repair pathway)	rs1052133, rs2072668, rs2304277, rs125701	5[34–36, 38, 39]	No
***MTHFR***	Methylenetetrahydrofolate reductase	Converts dietary folate is converted into 5-methyltetrahydrofolate, and controls serum homocysteine concentration	rs3737967, rs1801131, rs1801133, rs9651118	3[40–42]	No
***EPHA2***	Eph-receptor tyrosine kinase-type A2	Member of the Eph subfamily of receptor tyrosine kinases	rs7543472, rs11260867, rs7548209, rs3768293, rs6603867, rs6678616, rs477558, rs3754334, rs707455	4[43–46]	Yes[6]
***EFNA5***	Ephrin-A5	Receptor protein-tyrosine kinases involved in a variety of biological processes	c.668C>T (rs201008479), c.102C>T (rs199980747), c.–27C>G (rs200187971)	1[47]	No
***APOE***	Apolipoprotein E	Transporter of lipids and cholesterol	rs7412, rs429358	3[48–50]	No^a^
***KLC1***	Kinesin light chain 1	Kinesin-mediated cargo vesicle transport	rs8702, rs7154572, rs7150141, rs12432994, rs8007903, rs2403205, rs4900590, rs3212102, rs3212079	4[50–53]	No^a^
***HSF4***	Heat shock transcription factor 4	Regulator of the expression of several heat shock protein (HSP) genes	Copy number variation	1[54]	No
***GJA8***	Gap junction protein-alpha 8	Connexin50, a gap junction protein in the eye lens	rs1495960, rs9437983	1[55]	No
***FTO***	Fat mass and obesity-associated gene	Management of energy homeostasis, nucleic acid demethylation, and the regulation of body fat masses by lipolysis	rs9939609, rs9939973, rs9940128, rs1421085, rs1121980, rs7193144, rs17817449, rs8050136, rs9926289	2[26, 56]	No
***GALK1***	Galactokinase	Phosphorylates galactose to form galactose-1-phosphate, help making UDP-glucose, glycolipids and glycoproteins	c.252G->A, c.315G->A, c.615C->G, IVS4+34G->A, c.884G->A, c.1076T->C, c.1119G->A, IVS7+43C->T (rs743554)	1[57]	No
***MIP***	Major intrinsic protein of lens fiber	The most abundant junctional membrane protein in the mature lens	rs2269348, rs61759527, c.-4T>C, rs77163805, rs74641138, rs35033450, and rs36032520	1[58]	No
***IFN-G***	Interferon-gamma	Up regulate the first rate-limiting enzyme (IDO) in the tryptophan catabolism, which produces UV filters	+874(T/A)	1[59]	No
***IDO***	Indoleamine 2, 3-dioxygenase	The first rate limiting enzyme involved in the tryptophan catabolism which results in the production of UV filters	c.422+90G -> A (rs4613984)	1[60]	No
***NFE2L2***	Nuclear factor, erythroid 2 like 2	Regulator of antioxidant stress response	rs16865105, rs7557529, rs2886161, rs1806649, rs2001350, rs10183914, rs2706110, rs13035806	1[61]	No
***KEAP1***	Kelch like ECH associated protein 1	Regulator of antioxidant stress response	rs1048290, rs11085735 and rs1048287	1[61]	No
***UCHL1***	Ubiquitin carboxyl-terminal esterase L1	De-ubiquitinating enzyme with important functions in recycling of ubiquitin	c.53C ->A (rs5030732)	1[62]	No
***EZR***	Ezrin	A member of the ezrin/radixin/moesin (ERM) protein family, plays a crucial role in the development of the lens as a plasma membrane—cytoskeleton linker	rs5881286, rs2242318, rs144581330	1[63]	No
***HSP70***	70 kDa heat shock protein	Controls cellular responses to stress and apoptosis	HSPA1A Codon 190, HSPA1B Codon 1267, HSPA1L Codon 2437	1[64]	No
***TDRD7***	Tudor domain-containing protein 7	Component of RNA granule that control mRNA degradation, stabilization and subcellular localization	rs1462091, rs11793735, rs10981985, rs2045732, rs1462089	1[65]	No
***FABP2***	Fatty acid-binding protein-2	A protein expressed in enterocytes and is responsible for the absorption of long-chain fatty acids	Codon 54 (rs1799883)	1[66]	No
***PPARG2***	Peroxisome proliferator-activated receptor gama2	Ligand-activated transcription factor in the nuclear hormone receptor superfamily related to retinoid, steroid and thyroid hormone receptors	Codon 12	1[66]	No
***ESR***	Estrogen receptor	Estrogen receptor	ESR1: rs2234693, rs9340799; ESR2: rs4986938, rs1256031	1[67]	No
***CYP***	Cytochrome P450	Biosynthesis and bioavailability of multiple chemicals	CYP17A1: rs743572; CYP19A1: rs10046; CYP1A1: rs1048943	1[67]	No
***COMT***	Catechol-O-methyltransferase	Major degradative pathway of the catecholamine transmitters	rs4680	1[67]	No
***PSEN1***	Presenilin 1	Mutations of which were identified as causative of Alzheimer disease	rs165932, rs7523	1[50]	No

^a^ Although these genes had ≥3 related association studies, each of their polymorphisms was reported in less than three studies. Therefore, they were not chosen for meta-analysis.

### Inclusion of studies for meta-analysis

On the basis of the comprehensive review, three polymorphisms in two genes were extracted (8-oxoguanine DNA glycosylase-1 [*OGG1*]-rs1052133; methylenetetrahydrofolate reductase NADPH [*MTHFR*]-rs1801131, rs1801133), which were new for meta-analysis or the previous meta-analysis needed to be updated, and had sufficient genotype data in ≥3 studies.

**Fig 1:**
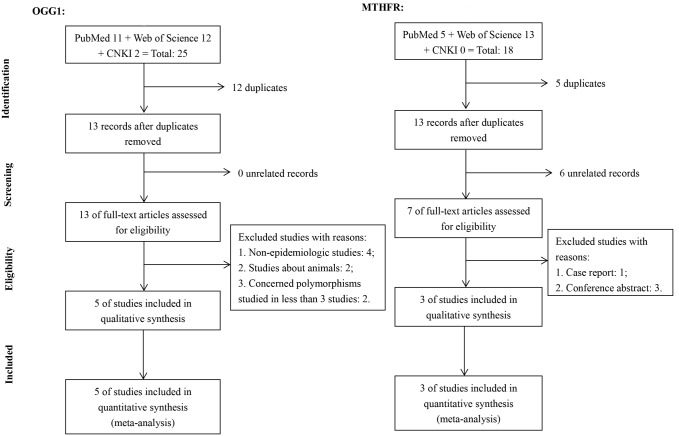
PRISMA flow diagram.

For the two genes, we identified a total of 43 records (*OGG1*: 25; *MTHFR*: 18). After removing 17 duplicates, we evaluated 26 records (*OGG1*: 13; *MTHFR*: 13) and excluded 6 unrelated records (*OGG1*: 0; *MTHFR*: 6). Among the 20 records remained (*OGG1*: 13; *MTHFR*: 7), 12 studies were excluded after full-text assessed for different reasons. Finally, eight studies (*OGG1*: 5; *MTHFR*: 3) were included in the qualitative synthesis and meta-analysis. More details can be found in Fig 1. Their major characteristics and Hardy-Weinberg equilibrium (HWE) test results are listed and compared in Table 2.

**Table 2:** Characteristics of the studies included in meta-analysis.

First author	Year	Sample size (case/control)	Age ^a^ (mean ± SD)	Gender ^b^ (Male %)	Case diagnosis	Control	Article language	Population ethnicity	HWE test (p value)	Quality score ^c^
**Included studies for *OGG1* (rs1052133)**										
**Zhang, Y.[**34**]**	2012	415, 386	65.77±6.49	52.3	ARC (cortical, nuclear, posterior subcapsular, mixed)	Disease-free volunteers	English	Chinese	0.3518	6
**Jiang, S.[**38**]**	2013	504, 244	60.2±5.7	47.1	ARC (cortical, nuclear, posterior subcapsular, mixed)	Healthy eyes and no systemic disease	English	Chinese	0.1328	8
**Gharib, A. F.[**35**]**	2014	150, 50	67.83±5.54	44.0	ARC (cortical, nuclear, posterior subcapsular)	Normal ocular examination	English	Egyptian	1.0000	6
**Wang, C.[**36**]**	2015	402, 813	67.45±7.01	49.8	ARC (subtypes not mentioned)	Without ARC and other age-related ocular diseases	English	Chinese	0.4688	7
**Wang, S.[**68**]**	2015	360, 392	66±6	54.8	ARC (cortical, nuclear, posterior subcapsular)	Without cataract and systemic diseases	Chinese	Chinese	0.2012	8
**Included studies for *MTHFR* (rs1801131, rs1801133)**										
**Zetterberg, M.[**40**]**	2005	502, 187	65.8±6.9	27.3	ARC (cortical, nuclear, posterior subcapsular, mixed)	Without cataract, uveitis and glaucoma	English	Caucasian	0.1601, 0.2458 ^d^	6
**Wang, X.[**41**]**	2015	502, 890	67.1±11.1	47.6	ARC (cortical, nuclear, posterior subcapsular, mixed)	Without cataract, other eye diseases and systemic diseases	English	Chinese	0.6537, 0.2696	7
**Tan, A. G.[**42**]**	2016	130, 627	65.3±6.9	46.6	ARC (cortical)	Without cortical cataract	English	Caucasian	0.8573, 0.2305	9

^a^ The mean age of control group.

^b^ The percentage of males in control group.

^c^ The quality of studies was assessed by Newcastle-Ottawa Scale (NOS), the quality score of which ranges from 0 (worst) to 9 (best).

^d^ The first number is for rs1801131, and the second for rs1801133.

### Quality assessment

Quality assessments by the NOS scores of the included observational studies are listed in Table 2. All eight eligible studies in the meta-analysis yielded scores ≥6, indicating relatively high methodological quality.

### Genetic associations of the *OGG1* gene with ARC

**Fig 2:**
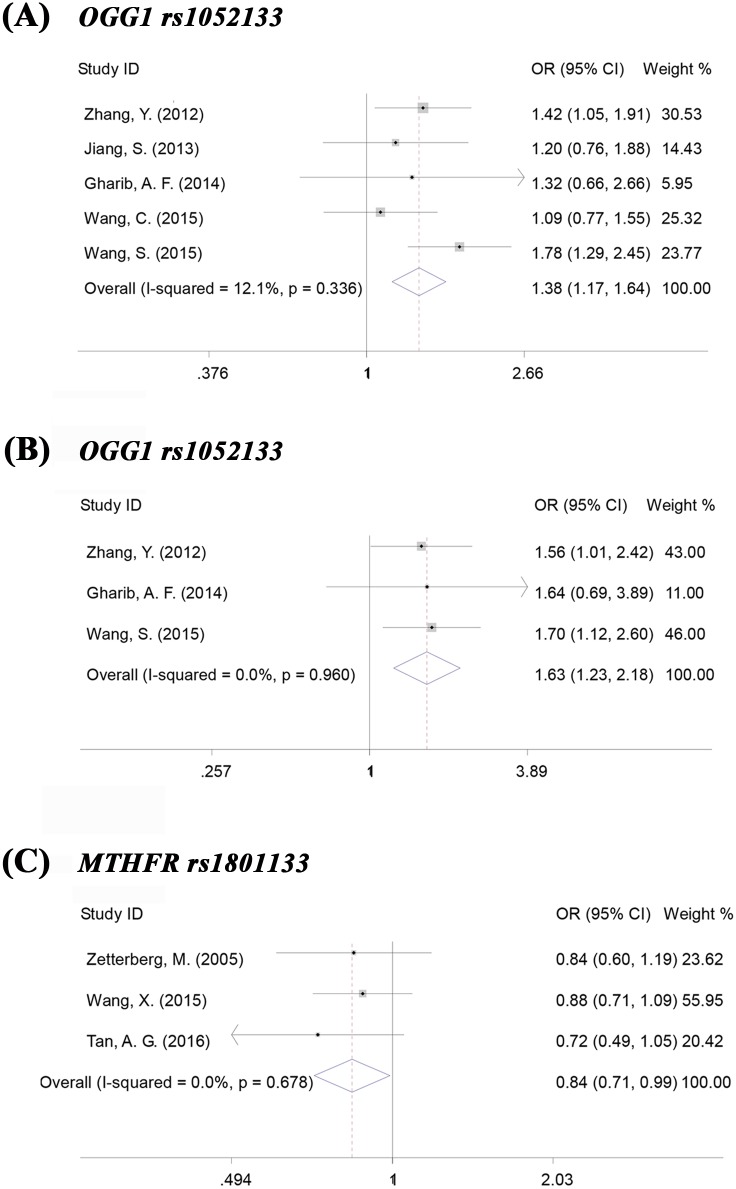
Forest plots for the association analysis of *OGG1* and *MTHFR* genes with age-related cataract. (A) *OGG1* rs1052133, association analysis of all cases in codominant model *CG* vs *CC*. (B) *OGG1* rs1052133, association analysis of cortical cases in codominant model *CG* vs *CC*. (C) *MTHFR* rs1801133, association analysis of all cases in overdominant model *CC+TT* vs *CT*.

**Fig 3:**
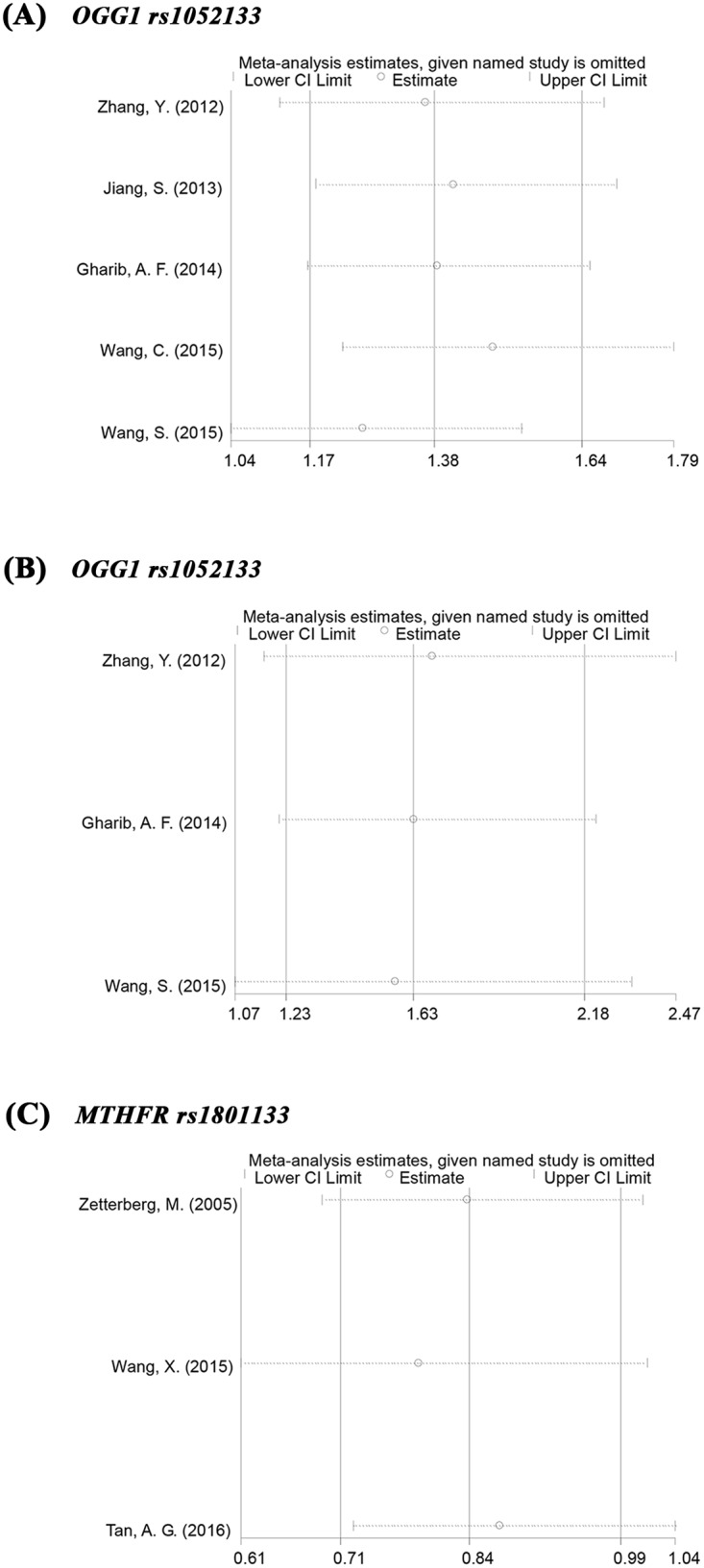
Sensitivity analysis for the association of *OGG1* and *MTHFR* genes with age-related cataract. (A) *OGG1* rs1052133, sensitivity analysis of all cases in codominant model *CG* vs *CC*. (B) *OGG1* rs1052133, sensitivity analysis of cortical cases in codominant model *CG* vs *CC*. (C) *MTHFR* rs1801133, sensitivity analysis of all cases in overdominant model *CC+TT* vs *CT*.

**Fig 4:**
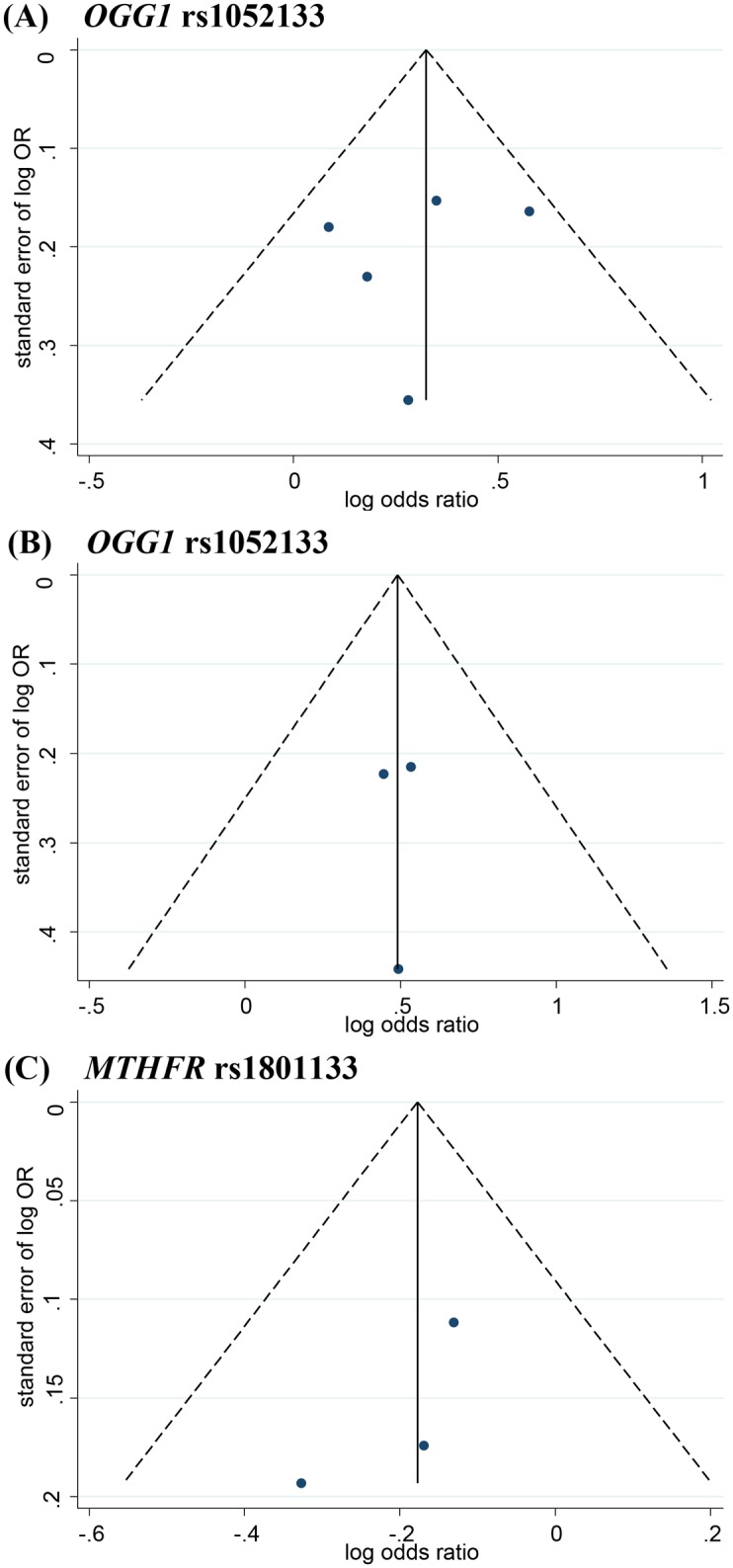
Funnel plot of the association of *OGG1* and *MTHFR* genes with age-related cataract. (A) *OGG1* rs1052133, funnel plot of all cases in codominant model *CG* vs *CC*. (B) *OGG1* rs1052133, funnel plot of cortical cases in codominant model *CG* vs *CC*. (C) *MTHFR* rs1801133, funnel plot of all cases in overdominant model *CC+TT* vs *CT*.

We used a model-free approach (details provided in Methods) to identify the best-fit genetic model. A codominant model was suggested for a λ value of 0.5. This approach revealed that the *GG* vs. *CC* genotype (OR = 1.925; 95%CI, 1.181–3.136; *p* = 0.009; I^2^ = 76.3%; Egger’s test, *p* = 0.469) and the *CG* vs. *CC* genotype (OR = 1.384; 95%CI, 1.171–1.636; *p*<0.001; I^2^ = 12.1%; Egger’s test, *p* = 0.613) were both significantly associated with an increased risk for ARC (forest plot shown in Fig 2A; sensitivity analysis in Fig 3A; funnel plot in Fig 4A). Subgroup analysis indicated that the significant associations also existed in most of the subtypes based on population ethnicity, article language and cataract morphology (see Table 3 for details). The significant associations were consistently found in some other genetic models, for example, the allelic, dominant, and recessive models for all cases and cortical subtype (for more details, refer to S1 Table). Substantial heterogeneity (I^2^>75%) existed in the overall analysis of the allelic, recessive and codominant (*GG* vs. *CC*) models. Regardless, the heterogeneity could be notably controlled in the subgroup analysis by cataract morphology, which represented the preponderant source of the heterogeneity.

**Table 3:** Meta-analysis for association of *OGG1* polymorphism (rs1052133) with age-related cataract.

Groups	N ^a^	Genetic model ^b^	Statistical method ^c^	I^2^	p_h_ ^d^	OR(95%CI)	p ^e^
**All**	5	Co-dominant (GG vs CC)	Random	76.3%	0.002	**1.925 (1.181, 3.136)**	0.009
	5	Co-dominant (CG vs CC)	Fixed	12.1%	0.336	**1.384 (1.171, 1.636)**	0.000
**Population ethnicity**							
Chinese	4	Co-dominant (GG vs CC)	Random	80.4%	0.002	**1.790 (1.080, 2.968)**	0.024
	4	Co-dominant (CG vs CC)	Fixed	33.9%	0.209	**1.388 (1.168, 1.648)**	0.000
Egyptian	1	Co-dominant (GG vs CC)	/	/	/	**4.571 (1.015, 20.592)**	0.048
	1	Co-dominant (CG vs CC)	/	/	/	1.325 (0.660, 2.659)	0.429
**Article language**							
English	4	Co-dominant (GG vs CC)	Random	54.5%	0.086	**1.558 (1.024, 2.371)**	0.039
	4	Co-dominant (CG vs CC)	Fixed	0.0%	0.726	**1.260 (1.036, 1.533)**	0.021
Chinese	1	Co-dominant (GG vs CC)	/	/	/	**3.207 (2.089, 4.925)**	0.000
	1	Co-dominant (CG vs CC)	/	/	/	**1.780 (1.291, 2.455)**	0.000
**Cataract morphology**							
Cortical	3	Co-dominant (GG vs CC)	Fixed	0.0%	0.505	**3.149 (2.069, 4.792)**	0.000
	3	Co-dominant (CG vs CC)	Fixed	0.0%	0.960	**1.635 (1.228, 2.176)**	0.001
Nuclear	3	Co-dominant (GG vs CC)	Fixed	0.0%	0.739	**1.911 (1.184, 3.083)**	0.008
	3	Co-dominant (CG vs CC)	Fixed	0.0%	0.886	**1.459 (1.085, 1.961)**	0.012
Posterior subcapsular	3	Co-dominant (GG vs CC)	Fixed	0.0%	0.660	**1.817 (1.026, 3.217)**	0.041
	3	Co-dominant (CG vs CC)	Fixed	0.0%	0.894	1.341 (0.939, 1.916)	0.106

^a^ N: The number of included studies.

^b^ Genetic model in this table was suggested by a model-free approach provided in methods. Results of other genetic models is shown in S1 Table.

^c^ If I^2^<50%, the fixed-effects model was used, otherwise, the random-effects model was used.

^d^ ph: p value of heterogeneity chi-squared test.

^e^ p: p value of test of OR = 1.

### Genetic associations of the *MTHFR* gene with ARC

Both *MTHFR* genetic polymorphisms, *C677T* (rs1801133) and *A1298C* (rs1801131), suggested the overdominant model by the λ calculation. The results of the suggested overdominant model are shown in Table 4. Additionally, the results of other genetic models are shown in S2 Table of the supplementary information.

**Table 4:** Meta-analysis for association of *MTHFR* polymorphisms (rs1801131, rs1801133) with age-related cataract.

Groups	N ^a^	Genetic model ^b^	Statistical method ^c^	I^2^	p_h_ ^d^	OR(95%CI)	p ^e^
**rs1801131 (A1298C)**							
All	3	Overdominant (AA+CC vs AC)	Fixed	4.5%	0.351	1.181 (0.991, 1.408)	0.063
Cataract morphology							
Cortical	3	Overdominant (AA+CC vs AC)	Fixed	0.0%	0.538	1.129 (0.900, 1.417)	0.294
Nuclear	2	Overdominant (AA+CC vs AC)	Random	60.2%	0.113	1.062 (0.632, 1.785)	0.821
Posterior subcapsular	2	Overdominant (AA+CC vs AC)	Fixed	0.0%	0.603	1.302 (0.909, 1.864)	0.150
Mixed	2	Overdominant (AA+CC vs AC)	Fixed	34.8%	0.216	**1.517 (1.113, 2.067)**	0.008
**rs1801133 (C677T)**							
All	3	Overdominant (CC+TT vs CT)	Fixed	0.0%	0.678	**0.838 (0.710, 0.989)**	0.036
Cataract morphology							
Cortical	3	Overdominant (CC+TT vs CT)	Fixed	0.0%	0.865	**0.731 (0.566, 0.945)**	0.017
Nuclear	2	Overdominant (CC+TT vs CT)	Fixed	0.0%	0.672	1.086 (0.714, 1.651)	0.699
Posterior subcapsular	2	Overdominant (CC+TT vs CT)	Fixed	0.0%	0.671	0.819 (0.546, 1.227)	0.332
Mixed	2	Overdominant (CC+TT vs CT)	Fixed	0.0%	0.753	0.848 (0.587, 1.225)	0.380
**Combined genotype of rs1801131 (A1298C) and rs1801133 (C677T)**							
**All**	2	677CC/1298AC vs 677CC/1298AA ^f^	Random	79.0%	0.029	0.505 (0.227, 1.128)	0.096
**Cataract morphology**							
Cortical	2	677CC/1298AC vs 677CC/1298AA	Fixed	0.0%	0.614	**0.483 (0.279, 0.836)**	0.009
Nuclear	2	677CC/1298AC vs 677CC/1298AA	Random	73.7%	0.051	0.653 (0.227, 1.873)	0.428
Posterior subcapsular	2	677CC/1298AC vs 677CC/1298AA	Fixed	0.0%	0.432	**0.465 (0.239, 0.906)**	0.025
Mixed	2	677CC/1298AC vs 677CC/1298AA	Random	90.8%	0.001	0.355 (0.064, 1.968)	0.236

^a^ N: The number of included studies.

^b^ Genetic model in this table was suggested by a model-free approach provided in methods. Results of other genetic models is shown in S2 Table.

^c^ If I^2^<50%, the fixed-effects model was used, otherwise, the random-effects model was used.

^d^ p_h_: p value of heterogeneity chi-squared test.

^e^ p: p value of test of OR = 1.

^f^ the wild genotype combination *677CC/1298AA* is used as reference in the association analysis of combined genotype.

### *C677T* (rs1801133)

The CC+TT vs. CT genotype (OR = 0.838; 95%CI, 0.710–0.989; p = 0.036; I^2^ = 0.0%; Egger’s test, p = 0.373) may be associated with a decreased risk for ARC (forest plot shown in Fig 2C, sensitivity analysis shown in Fig 3C; funnel plot shown in Fig 4C). However, in the subgroup analysis, this association was found for only the cortical subtype (OR = 0.731; 95%CI, 0.566–0.945; p = 0.017; I^2^ = 0.0%; Egger’s test, p = 0.599).

Regarding other genetic models, associations were found in the dominant and codominant models. The *CT+TT* vs. *CC* genotype (OR = 1.313; 95%CI, 1.104–1.562; *p* = 0.002; I^2^ = 44.6%; Egger’s test, *p* = 0.884) and *CT* vs. *CC* genotype (OR = 1.317; 95%CI, 1.095–1.584; *p* = 0.003; I^2^ = 0.0%; Egger’s test, *p* = 0.987) were recognized susceptible to ARC. However, the same associations existed in only the cortical subtype in subgroup analysis (for more details, refer to S2 Table). No substantial heterogeneity was found in the associations we identified for this locus.

### *A1298C* (rs1801131)

In the suggested overdominant model, no association was found in the overall and subgroup analyses of cortical, nuclear and posterior subcapsular morphology. However, in subgroup analysis of mixed morphology, the AA+CC vs. AC genotype (OR = 1.517; 95% CI, 1.113–2.067; p = 0.008; I^2^ = 34.8%) was found to be possibly associated with an increased risk for ARC.

Moreover, other genetic models revealed no association in overall and subgroup analyses of cortical, nuclear and posterior subcapsular morphology. For mixed morphology, the *AC+CC* vs. *AA* genotype (OR = 0.692; 95%CI, 0.513–0.932; *p* = 0.015; I^2^ = 28.0%) and *AC* vs. *AA* genotype (OR = 0.657; 95%CI, 0.480–0.900; *p* = 0.009; I^2^ = 37.7%) were both associated with decreased risk for ARC. No substantial heterogeneity was observed in the associations we assessed for this locus.

### Combined genotypes

Since the association analysis of the MTHFR gene concerned 2 SNPs (C677T and A1298C), we were interested in the genetic effect of their combined genotype on ARC susceptibility. The wild genotype combination 677CC/1298AA was used as the reference group and the pooled ORs and 95% CIs of other combined genotypes were calculated.

The *677CC/1298AC* combination was observed to be a protective factor in cortical cataract (OR = 0.483; 95%CI, 0.279–0.836; *p* = 0.009; I^2^ = 0.0%) and posterior subcapsular cataract (OR = 0.465; 95%CI, 0.239, 0.906; *p* = 0.025; I^2^ = 0.0%). More details are shown in Table 4. Other genotype combinations showed no association (results shown in S2 Table in the supplementary information). No heterogeneity was found for the associations we studied.

### Publication bias and sensitivity analysis

In the sensitivity analysis for all the genetic associations, the ORs were not substantially altered after removing any single studies. However, the following major associations were not robust because their p values were greater than 0.05 after removing one study: (1) *MTHFR*-rs1801131, mixed morphology subgroup, *AA+CC* vs. *AC*, after removing the study of Zetterberg, M.; (2) *MTHFR*-rs1801133, *CC+TT* vs. *CT*, after removing any one of the included studies and (3) *MTHFR*-rs1801133, cortical morphology subgroup, *CC+TT* vs. *CT*, after removing the study of Tan, A. G. Egger’s test *p*>0.05 for the main estimates indicated insignificant publication bias.

## Discussion

### Genes and loci most scrutinized in previous association studies regarding ARC

Environmental and genetic factors have been confirmed contributing to the pathogenesis of ARC [69, 70]. Genetic polymorphism has been recognized as a component of genetic risk for ARC and many studies have been conducted to identify the associations between genetic polymorphisms and ARC susceptibility [5–11]. In our systematic review, we summarized the genes/loci that have been studied by other investigators for the first time.

One intense area of study involves the genes of antioxidant enzymes that have roles in cellular defence mechanisms against oxidative stress, such as glutathione S transferase and superoxide dismutase (*SOD*). Oxidative stress has been well accepted as associated with age-related cataract (ARC) pathogenesis[71]. Specifically, the generation of excessive reactive oxygen species (ROS) leads to the abnormal degradation, cross-linking and aggregation of lens proteins, thus contributes to ARC genesis[72].

With impaired balance between the oxidative and antioxidative systems, DNA is damaged by accumulated ROS. Moreover, the DNA damage in the lens epithelium has been demonstrated to be associated with cataractogenesis [73, 74]. Therefore, another robust topic in previous association analysis has concerned the DNA repair enzyme genes, such as xeroderma pigmentosum complementation group D (*XPD*), and X-ray cross-complementing group 1 (*XRCC1*). For more details, refer to Table 1.

### Meta-analyzed genes and loci

Basic on this review, we selected the three polymorphisms of the two different genes (*OGG1*-rs1052133; *MTHFR*-rs1801131, rs1801133) that needed to be newly or updated meta-analyzed to undertake a quantitative synthesis. Among them, the *OGG1* gene encodes 8-oxoguanine glycosylase-1, a DNA repair enzyme of the base excision repair pathway that repairs oxidative DNA damage [75, 76]. Methylenetetrahydrofolate reductase, encoded by the *MTHFR* gene, controls serum homocysteine concentration, which has been considered associated with ARC susceptibility [77, 78].

Many associations were revealed from the five polymorphisms. Regarding rs1052133 in the *OGG1* gene, the *CG* and *GG* genotypes were both found risky for cataractogenesis, with approximately 1.4-fold and 1.9-fold increased risks, respectively. Alternatively, wild genotype *CC* was protective.

Regarding the *MTHFR* gene, the *CC+TT* genotype of rs1801133 was found to be protective. In contrast, the CT genotype was shown to have an adverse effect. However, the *AA+CC* genotype of rs1801131 indicated higher risk for mixed morphology cataract susceptibility. Haplotype analysis revealed that the combination of *677CC/1298AC* is protective against cortical cataract and posterior subcapsular cataract, and carries approximately a 0.5-fold decreased risk compared with the wild genotype combination (*677CC/1298AA*).

For most of our major conclusions, no substantial heterogeneity was found, which implied sound quality and consistent methodological design of the included studies. Differences in cataract morphology constitution may be the main source of substantial heterogeneity in most cases. Other potential sources of heterogeneity may derive from some uncontrolled confounding factors, for example, slightly different exclusion criteria of cases and controls, different mean ages and smoking status. Sensitivity analysis revealed that the genetic associations concerning the *MTHFR* gene were not as robust as the estimates for the other genes, which might be explained by the limited number of original articles.

A recently published study by Zhang et al [79] also examined the association of *OGG1* polymorphism with age-related cataract (ARC) and revealed *OGG1* polymorphism as a potential risk factor for ARC, in consistent with our findings. However, several discrepancies concerning analytic methodology and research findings could be found in between: (1) our literature search is more thorough, contains two more articles that double the number of cases and controls, thus, provide greater power to our conclusions; (2) we found new association between *G* allele and increased risk of age-related cataract (ARC) in allelic model (*p* = 0.008, shown in S1 Table); (3) another new association was found between *GG* genotype and higher ARC susceptibility, when compared with *CC* genotype in codominant model (*p* = 0.009, shown in Table 3). (4) we also found association in *CG* vs. *CC* genotype and *GG+CG* vs. *CC* genotype in nuclear subgroup in addition to cortical subtype; and (5) we used one more genetic model, the overdominant model, to gain a more comprehensive understanding of the association.

### Study strengths and limitations

This systematic review and meta-analysis is the first to provide a relatively thorough summary of the genes/loci involved in previous association studies of ARC. The *MTHFR* gene and its two polymorphisms were also meta-analyzed for the first time. The results reveal that to a certain degree, all these three genetic polymorphisms and two genes (*OGG1*-rs1052133; *MTHFR*-rs1801131, rs1801133) are associated with ARC susceptibility and may help identify high-risk populations in the future.

The review was undertaken using a meticulous methodology. Strict inclusion criteria were adopted. Every included study was of high quality and achieved Hardy-Weinberg equilibrium. A model-free approach was used to suggest a best-fit genetic model, results of which are shown in Tables 3 and 4. Notwithstanding, other genetic models were also used to reach more comprehensive conclusions (results shown in S1 and S2 Tables).

The main limitation of the meta-analysis is that the polymorphisms of the MTHFR gene were studied in a limited number of original articles, which rendered some revealed associations less robust in the sensitivity analysis. Other limitations pertained to the published studies and to our review. Firstly, age-related cataract is a multifactorial disease. Other confounding factors such as ultraviolet light exposure and smoking may also influence the association analysis. However, the included studies did not provide detailed records of these confounding factors. Thus, the associations we found in our review may be strengthened or weakened by these confounders. We anticipate that future studies will direct greater attention to these influences when possible. Secondly, the ethnicity involved in our review is limited. For example, four of the five included studies of rs1052133 in *OGG1* gene focused on the Han Chinese population, which restricts the applicability of our conclusions to a certain ethnicity. The emergence of future studies that focus on other ethnicities will facilitate the determination of the associations in other populations. Thirdly, the precise mechanisms of the genetic effects we observed remain unknown. Studies of underlying mechanisms are needed.

In summary, we consider these polymorphisms to represent new candidate biomarkers for high-risk ARC population. However, additional original research with larger sample sizes, high quality, and broader ethnicity coverage remain anticipated.

## Supporting information

S1 Appendix Database search. (DOC)

S2 Appendix Excluded articles with reasons. (DOCX)

S1 Table Association analysis of *OGG1* polymorphism (rs1052133) with age-related cataract in other genetic models. (DOC)

S2 Table Association analysis of *MTHFR* polymorphisms (rs1801131, rs1801133) with age-related cataract in other genetic models. (DOC)

S3 Table Genotype data extracted from included studies. (DOCX)

S1 Checklist PRISMA checklist. (DOC)

S2 Checklist PLOS ONE meta-analysis on genetic association studies checklist. (DOCX)
