# Supplementary material for: Association of OGG1 and MTHFR polymorphisms with age-related cataract: A systematic review and meta-analysis
Source: PLoS One. 2017 Mar 2;12(3):e0172092. doi: 10.1371/journal.pone.0172092 (PMC5333819; doi:10.1371/journal.pone.0172092)
Supplement: S1 Appendix — (DOC) [file pone.0172092.s001.doc]

**S1 Appendix. Database search.**

**(our literature search was completed before June 26, 2016)**

**General literature search:**

Search strategy for PubMed:

("polymorphism, genetic"[MeSH Terms] OR ("polymorphism"[All Fields] AND "genetic"[All Fields]) OR "genetic polymorphism"[All Fields] OR "polymorphism"[All Fields]) AND ("cataract"[MeSH Terms] OR "cataract"[All Fields])

Search strategy for Web of Science:

TS=(cataract) AND TS=(polymorphism)

**Literature search for OGG1 gene:**

Search strategy for PubMed:

(OGG1[All Fields] OR (("8-hydroxyguanine"[Supplementary Concept] OR "8-hydroxyguanine"[All Fields] OR "8 oxoguanine"[All Fields]) AND glycosylase-1[All Fields])) AND ("cataract"[MeSH Terms] OR "cataract"[All Fields])

Search strategy for Web of Science:

TS=(OGG1 OR 8-oxoguanine glycosylase-1) AND TS=cataract

Search strategy for China National Knowledge Internet (CNKI):

(8-oxoguanine glycosylase-1[in Chinese] OR OGG1) AND (cataract[in Chinese] OR cataract)

**Literature search for MTHFR gene:**

Search strategy for PubMed:

(("methylenetetrahydrofolate reductase (nadph2)"[MeSH Terms] OR ("methylenetetrahydrofolate"[All Fields] AND "reductase"[All Fields] AND "(nadph2)"[All Fields]) OR "methylenetetrahydrofolate reductase (nadph2)"[All Fields] OR "mthfr"[All Fields]) OR ("methylenetetrahydrofolate reductase (nadph2)"[MeSH Terms] OR ("methylenetetrahydrofolate"[All Fields] AND "reductase"[All Fields] AND "(nadph2)"[All Fields]) OR "methylenetetrahydrofolate reductase (nadph2)"[All Fields] OR ("methylenetetrahydrofolate"[All Fields] AND "reductase"[All Fields]) OR "methylenetetrahydrofolate reductase"[All Fields])) AND ("cataract"[MeSH Terms] OR "cataract"[All Fields])

Search strategy for Web of Science:

TS=(MTHFR OR methylenetetrahydrofolate reductase) AND TS=cataract

Search strategy for China National Knowledge Internet (CNKI):

(methylenetetrahydrofolate reductase[in Chinese] OR MTHFR) AND (cataract[in Chinese] OR cataract)
