# Supplementary material for: Association of OGG1 and MTHFR polymorphisms with age-related cataract: A systematic review and meta-analysis
Source: PLoS One. 2017 Mar 2;12(3):e0172092. doi: 10.1371/journal.pone.0172092 (PMC5333819; doi:10.1371/journal.pone.0172092)
Supplement: S2 Appendix — (DOCX) [file pone.0172092.s002.docx]

**Excluded articles of *OGG1* gene (with reason):**

1. Zhang Y, OuYang S, Zhang L, Tang X, Son Z, Liu P. Oxygen-induced changes in mitochondrial DNA and DNA repair enzymes in aging rat lens. Mechanisms of ageing and development. 2010;131(11-12):666-73.(Study about animals)

2. Zhang Y, Zhang L, Zhang L, Bai J, Ge H, Liu P. Expression changes in DNA repair enzymes and mitochondrial DNA damage in aging rat lens. Molecular vision. 2010;16(190):1754-63.(Study about animals)

3. Yang M, Su S, Zhou J, Zhu R, Qin B, Yang L, et al. Study on gene-gene, gene-environmental interactions of DNA repair genes related with age-related cataract. National Medical Journal of China. 2014;94(15):1147-51.(Concerned polymorphisms studied in less than 3 studies)

4. Su S, Yao Y, Zhu R, Liang C, Jiang S, Hu N, et al. The Associations between Single Nucleotide Polymorphisms of DNA Repair Genes, DNA Damage, and Age-Related Cataract: Jiangsu Eye Study. Investigative ophthalmology & visual science. 2013;54(2):1201-7. (Concerned polymorphisms studied in less than 3 studies)

5. Xu B, Kang L, Zhang G, Wu J, Zhu R, Yang M, et al. The Changes of 8-OHdG, hOGG1, APE1 and Pol beta in Lenses of Patients with Age-Related Cataract. Current eye research. 2015;40(4):378-85.(None-epidemiologic studies)

6. Wang Y, Li F, Zhang G, Kang L, Qin B, Guan H. Altered DNA Methylation and Expression Profiles of 8-Oxoguanine DNA Glycosylase 1 in Lens Tissue from Age-related Cataract Patients. Current eye research. 2015;40(8):815-21.(None-epidemiologic studies)

7. Kang L, Zhao W, Zhang G, Wu J, Guan H. Acetylated 8-oxoguanine DNA glycosylase 1 and its relationship with p300 and SIRT1 in lens epithelium cells from age-related cataract. Experimental eye research. 2015;135:102-8. (None-epidemiologic studies)

8. Li C, Guan H. Clinical study about relationship between human 8-oxoguanine DNA glycosylase-1 and age-related cataract. Journal of Clinical Ophthalmology. 2014(06):481-5. (None-epidemiologic studies)

**Excluded articles of *MTHFR* gene (with reason):**

1. Junemann AG, von Ahsen N, Kornhuber J, Ritter K, Naumann GOH, Bleich S. MTHFR C677T POLYMORPHISM IS A GENETIC RISK FACTOR FOR PRIMARY OPEN - ANGLE GLAUCOMA. ARVO Annual Meeting Abstract Search and Program Planner. 2003;2003:93.(Conference abstract)

2. Zetterberg H, Zetterberg M, Prince JA, Tasa G, Karlsson JOO, Blennow K. Methylenetetrahydrofolate reductase polymorphisms in human cataract. Investigative Ophthalmology & Visual Science. 2005;46. (Conference abstract)

3. Tan AG, Mitchell P, Rochtchina E, Kifley A, Flood V, Jun G, et al. Methylenetetrahydrofolate reductase polymorphisms, serum homocysteine and incident cortical cataract. Investigative Ophthalmology & Visual Science. 2015;56(7). (Conference abstract)

4. Erol M, Gayret OB, Yigit O, Cabuk KS, Toksoz M, Tiras M. A Case of Homocystinuria Misdiagnosed as Moyamoya Disease: A Case Report. Iranian Red Crescent medical journal. 2016;18(4).(Case report)
