# Supplementary material for: Association of OGG1 and MTHFR polymorphisms with age-related cataract: A systematic review and meta-analysis
Source: PLoS One. 2017 Mar 2;12(3):e0172092. doi: 10.1371/journal.pone.0172092 (PMC5333819; doi:10.1371/journal.pone.0172092)
Supplement: S1 Table — (DOC) [file pone.0172092.s003.doc]

**S1 Table. Association analysis of *OGG1* polymorphism (rs1052133) with age-related cataract in other genetic models.**

| **Groups** | **N ^a^** | **Genetic model ^b^** | **Statistical method ^c^** | **I^2^** | **p_h_ ^d^** | **OR(95%CI)** | **p ^e^** |
| --- | --- | --- | --- | --- | --- | --- | --- |
| **All** | 5 | Allele (G vs C) | Random | 80.9% | 0.000 | **1.397 (1.092, 1.788)** | 0.008 |
|  | 5 | Dominant (CG+GG vs CC) | Random | 53.9% | 0.070 | **1.498 (1.174, 1.910)** | 0.001 |
|  | 5 | Recessive (GG vs CG+CC) | Random | 76.4% | 0.002 | **1.581 (1.066, 2.346)** | 0.023 |
|  | 5 | Overdominant (CC+GG vs CG) | Fixed | 0.0% | 0.475 | 0.903 (0.788, 1.035) | 0.144 |
| **Population ethnicity** | | | | | | | |
| Chinese | 4 | Allele (G vs C) | Random | 84.7% | 0.000 | **1.347 (1.033, 1.757)** | 0.028 |
|  | 4 | Dominant (CG+GG vs CC) | Random | 65.0% | 0.035 | **1.473 (1.115, 1.947)** | 0.006 |
|  | 4 | Recessive (GG vs CG+CC) | Random | 79.7% | 0.002 | **1.489 (1.004, 2.208)** | 0.048 |
|  | 4 | Overdominant (CC+GG vs CG) | Fixed | 14.7% | 0.319 | 0.903 (0.786, 1.038) | 0.150 |
| Egyptian | 1 | Allele (G vs C) | / | / | / | **1.854 (1.073, 3.203)** | 0.027 |
|  | 1 | Dominant (CG+GG vs CC) | / | / | / | 1.685 (0.871, 3.262) | 0.121 |
|  | 1 | Recessive (GG vs CG+CC) | / | / | / | 4.125 (0.934, 18.213) | 0.061 |
|  | 1 | Overdominant (CC+GG vs CG) | / | / | / | 0.913 (0.461, 1.809) | 0.795 |
| **Article language** | | | | | | | |
| English | 4 | Allele (G vs C) | Random | 64.2% | 0.039 | **1.263 (1.023, 1.560)** | 0.030 |
|  | 4 | Dominant (CG+GG vs CC) | Fixed | 0.0% | 0.458 | **1.349 (1.122, 1.623)** | 0.001 |
|  | 4 | Recessive (GG vs CG+CC) | Random | 59.7% | 0.059 | 1.330 (0.938, 1.887) | 0.109 |
|  | 4 | Overdominant (CC+GG vs CG) | Fixed | 0.0% | 0.431 | 0.933 (0.799, 1.089) | 0.380 |
| Chinese | 1 | Allele (G vs C) | / | / | / | **1.868 (1.515, 2.304)** | 0.000 |
|  | 1 | Dominant (CG+GG vs CC) | / | / | / | **2.101 (1.557, 2.835)** | 0.000 |
|  | 1 | Recessive (GG vs CG+CC) | / | / | / | **2.361 (1.596, 3.492)** | 0.000 |
|  | 1 | Overdominant (CC+GG vs CG) | / | / | / | 0.806 (0.604, 1.077) | 0.145 |
| **Cataract morphology** | | | | | | | |
| Cortical | 3 | Allele (G vs C) | Fixed | 0.0% | 0.510 | **1.799 (1.478, 2.189)** | 0.000 |
|  | 3 | Dominant (CG+GG vs CC) | Fixed | 0.0% | 0.753 | **1.898 (1.453, 2.479)** | 0.000 |
|  | 3 | Recessive (GG vs CG+CC) | Fixed | 0.0% | 0.488 | **2.464 (1.672, 3.630)** | 0.000 |
|  | 3 | Overdominant (CC+GG vs CG) | Fixed | 0.0% | 0.858 | 0.781 (0.598, 1.018) | 0.068 |
| Nuclear | 3 | Allele (G vs C) | Fixed | 0.0% | 0.901 | **1.423 (1.151, 1.760)** | 0.001 |
|  | 3 | Dominant (CG+GG vs CC) | Fixed | 0.0% | 0.935 | **1.536 (1.161, 2.032)** | 0.003 |
|  | 3 | Recessive (GG vs CG+CC) | Fixed | 0.0% | 0.649 | **1.574 (1.004, 2.469)** | 0.048 |
|  | 3 | Overdominant (CC+GG vs CG) | Fixed | 0.0% | 0.901 | 0.761 (0.574, 1.009) | 0.057 |
| Posterior subcapsular | 3 | Allele (G vs C) | Fixed | 0.0% | 0.884 | **1.360 (1.053, 1.757)** | 0.019 |
|  | 3 | Dominant (CG+GG vs CC) | Fixed | 0.0% | 0.992 | **1.427 (1.020, 1.997)** | 0.038 |
|  | 3 | Recessive (GG vs CG+CC) | Fixed | 0.0% | 0.533 | 1.550 (0.905, 2.655) | 0.110 |
|  | 3 | Overdominant (CC+GG vs CG) | Fixed | 0.0% | 0.853 | 0.813 (0.579, 1.143) | 0.234 |

^a^ N: The number of included studies.

^b^ Genetic model in this table was suggested by a model-free approach provided in methods.

^c^ If I^2^<50%, the fixed-effects model was used, otherwise, the random-effects model was used.

^d^ p_h_: p value of heterogeneity chi-squared test.

^e^ p: p value of test of OR=1.
