# Supplementary material for: Association of OGG1 and MTHFR polymorphisms with age-related cataract: A systematic review and meta-analysis
Source: PLoS One. 2017 Mar 2;12(3):e0172092. doi: 10.1371/journal.pone.0172092 (PMC5333819; doi:10.1371/journal.pone.0172092)
Supplement: S2 Table — (DOC) [file pone.0172092.s004.doc]

**S2 Table. Association analysis of *MTHFR* polymorphisms (rs1801131, rs1801133) with age-related cataract in other genetic models.**

| **Groups** | **N ^a^** | **Genetic model ^b^** | **Statistical method ^c^** | **I^2^** | **p_h_ ^d^** | **OR(95%CI)** | **p ^e^** |
| --- | --- | --- | --- | --- | --- | --- | --- |
| **rs1801131 (A1298C)** | | | | | | | |
| **All** | 3 | Allele (C vs A) | Fixed | 21.3% | 0.281 | 0.951 (0.828, 1.092) | 0.477 |
|  | 3 | Dominant (AC+CC vs AA) | Fixed | 31.7% | 0.231 | 0.890 (0.750, 1.055) | 0.179 |
|  | 3 | Recessive (CC vs AC+AA) | Fixed | 0.0% | 0.496 | 1.180 (0.839, 1.661) | 0.342 |
|  | 3 | Codominant (CC vs AA) | Fixed | 2.5% | 0.359 | 1.083 (0.762, 1.538) | 0.656 |
|  | 3 | Codominant (AC vs AA) | Fixed | 19.4% | 0.289 | 0.853 (0.713, 1.020) | 0.081 |
| **Cataract morphology** | | | | | | | |
| Cortical | 3 | Allele (C vs A) | Fixed | 14.5% | 0.310 | 0.945 (0.791, 1.128) | 0.531 |
|  | 3 | Dominant (AC+CC vs AA) | Fixed | 15.3% | 0.307 | 0.903 (0.723, 1.126) | 0.364 |
|  | 3 | Recessive (CC vs AC+AA) | Fixed | 0.0% | 0.630 | 1.053 (0.689, 1.608) | 0.812 |
|  | 3 | Codominant (CC vs AA) | Fixed | 0.0% | 0.460 | 0.971 (0.628, 1.503) | 0.896 |
|  | 3 | Codominant (AC vs AA) | Fixed | 0.0% | 0.409 | 0.882 (0.699, 1.113) | 0.291 |
| Nuclear | 2 | Allele (C vs A) | Fixed | 0.0% | 0.961 | 1.115 (0.869, 1.429) | 0.392 |
|  | 2 | Dominant (AC+CC vs AA) | Fixed | 0.0% | 0.449 | 1.079 (0.796, 1.462) | 0.625 |
|  | 2 | Recessive (CC vs AC+AA) | Fixed | 30.7% | 0.230 | 1.479 (0.788, 2.775) | 0.223 |
|  | 2 | Codominant (CC vs AA) | Fixed | 0.0% | 0.361 | 1.425 (0.744, 2.731) | 0.286 |
|  | 2 | Codominant (AC vs AA) | Fixed | 34.6% | 0.216 | 1.036 (0.754, 1.423) | 0.828 |
| Posterior subcapsular | 2 | Allele (C vs A) | Fixed | 0.0% | 0.447 | 1.035 (0.787, 1.361) | 0.807 |
|  | 2 | Dominant (AC+CC vs AA) | Fixed | 0.0% | 0.809 | 0.908 (0.644, 1.280) | 0.581 |
|  | 2 | Recessive (CC vs AC+AA) | Random | 50.2% | 0.156 | 1.875 (0.779, 4.512) | 0.160 |
|  | 2 | Codominant (CC vs AA) | Fixed | 47.5% | 0.167 | 1.646 (0.860, 3.151) | 0.133 |
|  | 2 | Codominant (AC vs AA) | Fixed | 0.0% | 0.742 | 0.800 (0.553, 1.156) | 0.235 |
| Mixed | 2 | Allele (C vs A) | Fixed | 0.0% | 0.444 | 0.787 (0.614, 1.009) | 0.059 |
|  | 2 | Dominant (AC+CC vs AA) | Fixed | 28.0% | 0.239 | **0.692 (0.513, 0.932)** | 0.015 |
|  | 2 | Recessive (CC vs AC+AA) | Fixed | 0.0% | 0.929 | 1.117 (0.593, 2.107) | 0.732 |
|  | 2 | Codominant (CC vs AA) | Fixed | 0.0%, | 0.829 | 0.935 (0.488, 1.790) | 0.839 |
|  | 2 | Codominant (AC vs AA) | Fixed | 37.7% | 0.205 | **0.657 (0.480, 0.900)** | 0.009 |
| **rs1801133 (C677T)** | | | | | | | |
| **All** | 3 | Allele (T vs C) | Random | 64.3% | 0.061 | 1.162 (0.934, 1.446) | 0.177 |
|  | 3 | Dominant (CT+TT vs CC) | Fixed | 44.6% | 0.165 | **1.313 (1.104, 1.562)** | 0.002 |
|  | 3 | Recessive (TT vs CT+CC) | Random | 63.6% | 0.064 | 1.050 (0.685, 1.612) | 0.822 |
|  | 3 | Codominant (TT vs CC) | Random | 71.8% | 0.029 | 1.209 (0.712, 2.051) | 0.482 |
|  | 3 | Codominant (CT vs CC) | Fixed | 0.0% | 0.453 | **1.317 (1.095, 1.584)** | 0.003 |
| **Cataract morphology** | | | | | | | |
| Cortical | 3 | Allele (T vs C) | Fixed | 42.8% | 0.174 | 1.141 (0.945, 1.378) | 0.169 |
|  | 3 | Dominant (CT+TT vs CC) | Fixed | 15.3% | 0.307 | **1.331 (1.024, 1.731)** | 0.033 |
|  | 3 | Recessive (TT vs CT+CC) | Fixed | 37.6% | 0.201 | 0.924 (0.626, 1.363) | 0.689 |
|  | 3 | Codominant (TT vs CC) | Random | 51.6% | 0.127 | 1.089 (0.582, 2.039) | 0.790 |
|  | 3 | Codominant (CT vs CC) | Fixed | 0.0% | 0.545 | **1.404 (1.065, 1.852)** | 0.016 |
| Nuclear | 2 | Allele (T vs C) | Fixed | 0.0% | 0.368 | 0.780 (0.569, 1.068) | 0.121 |
|  | 2 | Dominant (CT+TT vs CC) | Fixed | 0.0% | 0.485 | 0.766 (0.505, 1.162) | 0.210 |
|  | 2 | Recessive (TT vs CT+CC) | Fixed | 0.0% | 0.437 | 0.659 (0.338, 1.284) | 0.220 |
|  | 2 | Codominant (TT vs CC) | Fixed | 0.0% | 0.384 | 0.603 (0.298, 1.219) | 0.159 |
|  | 2 | Codominant (CT vs CC) | Fixed | 0.0% | 0.599 | 0.829 (0.533, 1.289) | 0.405 |
| Posterior subcapsular | 2 | Allele (T vs C) | Fixed | 0.0% | 0.975 | 0.997 (0.739, 1.346) | 0.985 |
|  | 2 | Dominant (CT+TT vs CC) | Fixed | 0.0% | 0.825 | 1.104 (0.734, 1.660) | 0.636 |
|  | 2 | Recessive (TT vs CT+CC) | Fixed | 0.0% | 0.697 | 0.791 (0.423, 1.478) | 0.462 |
|  | 2 | Codominant (TT vs CC) | Fixed | 0.0% | 0.806 | 0.848 (0.436, 1.649) | 0.626 |
|  | 2 | Codominant (CT vs CC) | Fixed | 0.0% | 0.739 | 1.185 (0.770, 1.822) | 0.441 |
| Mixed | 2 | Allele (T vs C) | Random | 69.8% | 0.069 | 1.241 (0.727, 2.116) | 0.429 |
|  | 2 | Dominant (CT+TT vs CC) | Fixed | 39.2% | 0.200 | 1.246 (0.854, 1.818) | 0.255 |
|  | 2 | Recessive (TT vs CT+CC) | Random | 71.5% | 0.061 | 1.180 (0.447, 3.111) | 0.739 |
|  | 2 | Codominant (TT vs CC) | Random | 75.0% | 0.045 | 1.406 (0.413, 4.790) | 0.586 |
|  | 2 | Codominant (CT vs CC) | Fixed | 0.0% | 0.457 | 1.270 (0.848, 1.901) | 0.246 |
| **Combined genotype of rs1801131 (A1298C) and rs1801133 (C677T)** | | | | | | | |
| **All** | 2 | 677CT/1298AA vs 677CC/1298AA **^f^** | Random | 77.0% | 0.037 | 0.801 (0.399, 1.607) | 0.532 |
|  | 2 | 677CC/1298CC vs 677CC/1298AA | Fixed | 0.0% | 0.622 | 0.757 (0.416, 1.375) | 0.360 |
|  | 2 | 677CT/1298AC vs 677CC/1298AA | Random | 68.5% | 0.075 | 0.920 (0.475, 1.783) | 0.806 |
|  | 2 | 677TT/1298AA vs 677CC/1298AA | Random | 91.3% | 0.001 | 0.709 (0.182, 2.761) | 0.620 |
| **Cataract morphology** | | | | | | | |
| Cortical | 2 | 677CT/1298AA vs 677CC/1298AA | Random | 69.1% | 0.072 | 0.959 (0.433, 2.123) | 0.917 |
|  | 2 | 677CC/1298CC vs 677CC/1298AA | Fixed | 0.0% | 0.709 | 0.579 (0.236, 1.419) | 0.232 |
|  | 2 | 677CT/1298AC vs 677CC/1298AA | Random | 72.0% | 0.059 | 1.245 (0.499, 3.106) | 0.639 |
|  | 2 | 677TT/1298AA vs 677CC/1298AA | Random | 86.8% | 0.006 | 0.765 (0.166, 3.522) | 0.730 |
| Nuclear | 2 | 677CT/1298AA vs 677CC/1298AA | Random | 73.0% | 0.054 | 0.768 (0.293, 2.012) | 0.591 |
|  | 2 | 677CC/1298CC vs 677CC/1298AA | Fixed | 0.0% | 0.441 | 0.771 (0.308, 1.929) | 0.578 |
|  | 2 | 677CT/1298AC vs 677CC/1298AA | Random | 74.1% | 0.049 | 0.849 (0.279, 2.582) | 0.773 |
|  | 2 | 677TT/1298AA vs 677CC/1298AA | Random | 75.0% | 0.045 | 0.561 (0.147, 2.148) | 0.399 |
| Posterior subcapsular | 2 | 677CT/1298AA vs 677CC/1298AA | Fixed | 0.0% | 0.891 | 0.817 (0.487, 1.369) | 0.442 |
|  | 2 | 677CC/1298CC vs 677CC/1298AA | Fixed | 37.4% | 0.206 | 1.341 (0.588, 3.056) | 0.486 |
|  | 2 | 677CT/1298AC vs 677CC/1298AA | Fixed | 0.0% | 0.798 | 0.831 (0.449, 1.538) | 0.556 |
|  | 2 | 677TT/1298AA vs 677CC/1298AA | Fixed | 27.0% | 0.242 | 0.893 (0.485, 1.645) | 0.716 |
| Mixed | 2 | 677CT/1298AA vs 677CC/1298AA | Random | 73.3% | 0.053 | 0.622 (0.268, 1.444) | 0.270 |
|  | 2 | 677CC/1298CC vs 677CC/1298AA | Fixed | 0.0% | 0.533 | 0.523 (0.225, 1.216) | 0.132 |
|  | 2 | 677CT/1298AC vs 677CC/1298AA | Fixed | 0.0% | 0.491 | 0.596 (0.354, 1.003) | 0.051 |
|  | 2 | 677TT/1298AA vs 677CC/1298AA | Random | 90.3% | 0.001 | 0.701 (0.131, 3.750) | 0.678 |

^a^ N: The number of included studies.

^b^ Genetic model in this table was suggested by a model-free approach provided in methods.

^c^ If I^2^<50%, the fixed-effects model was used, otherwise, the random-effects model was used.

^d^ p_h_: p value of heterogeneity chi-squared test.

^e^ p: p value of test of OR=1.

^f^ the wild genotype combination 677CC/1298AA is used as reference in the association analysis of combined genotype.
