# Supplementary material for: Association of OGG1 and MTHFR polymorphisms with age-related cataract: A systematic review and meta-analysis
Source: PLoS One. 2017 Mar 2;12(3):e0172092. doi: 10.1371/journal.pone.0172092 (PMC5333819; doi:10.1371/journal.pone.0172092)
Supplement: S3 Table — (DOCX) [file pone.0172092.s005.docx]

**S3 Table. Genotype data extracted from included studies**

| Studies |  | Cases | | | | |  | | | Controls | | | | |
| --- | --- | --- | --- | --- | --- | --- | --- | --- | --- | --- | --- | --- | --- | --- |
| rs1052133 |  | CC | CG | GG | C | G | |  | CC | | CG | GG | C | G |
| Zhang, Y.[1] |  | 222 | 153 | 40 | 597 | 233 | |  | 247 | | 120 | 19 | 614 | 158 |
| Jiang, S.[2] |  | 72 | 222 | 210 | 366 | 642 | |  | 40 | | 103 | 101 | 183 | 305 |
| Gharib, A. F.[3] |  | 77 | 51 | 22 | 205 | 95 | |  | 32 | | 16 | 2 | 80 | 20 |
| Wang, C.[4] |  | 58 | 194 | 150 | 310 | 494 | |  | 132 | | 405 | 276 | 669 | 957 |
| Wang, S.[5] |  | 109 | 165 | 86 | 383 | 337 | |  | 187 | | 159 | 46 | 533 | 251 |
| rs1801133 |  | CC | CT | TT | C | T | |  | CC | | CT | TT | C | T |
| Zetterberg, M.[6] |  | 239 | 222 | 41 | 700 | 304 | |  | 89 | | 75 | 23 | 253 | 121 |
| Wang, X.[7] |  | 139 | 251 | 112 | 529 | 475 | |  | 312 | | 416 | 162 | 1040 | 740 |
| Tan, A. G.[8] |  | 48 | 64 | 18 | 160 | 100 | |  | 299 | | 258 | 70 | 856 | 398 |
| rs1801131 |  | CC | AC | AA | C | A | |  | CC | | AC | AA | C | A |
| Zetterberg, M.[6] |  | 41 | 193 | 268 | 275 | 729 | |  | 12 | | 87 | 88 | 111 | 263 |
| Wang, X.[7] |  | 25 | 143 | 334 | 193 | 811 | |  | 32 | | 262 | 596 | 326 | 1454 |
| Tan, A. G.[8] |  | 13 | 49 | 68 | 75 | 185 | |  | 71 | | 276 | 280 | 418 | 836 |

**References**

1. Zhang Y, Zhang L, Song Z, Sun DL, Liu HR, Fu SB, et al. Genetic polymorphisms in DNA repair genes OGG1, APE1, XRCC1, and XPD and the risk of age-related cataract. Ophthalmology. 2012;119(5):900-6.

2. Jiang S, Hu N, Zhou J, Zhang J, Gao R, Hu J, et al. Polymorphisms of the WRN gene and DNA damage of peripheral lymphocytes in age-related cataract in a Han Chinese population. Age (Dordrecht, Netherlands). 2013;35(6):2435-44.

3. Gharib AF, Dabour SA, Etewa RL, Fouad RA. Polymorphisms of DNA repair genes OGG1 and XPD and the risk of age-related cataract in Egyptians. Molecular vision. 2014;20:661-9.

4. Wang C, Lai Q, Zhang S, Hu J. Senile cataract and genetic polymorphisms of APE1, XRCC1 and OGG1. International Journal of Clinical and Experimental Pathology. 2015;8(12):16036-45.

5. Wang S, Wang C, He J, Hou L. Clinical study of the relation between OGG1 gene and age-related cataract. Shanxi Medical Journal. 2015(14):1639-41.(in Chinese)

6. Zetterberg M, Tasa G, Prince JA, Palmer M, Juronen E, Veromann S, et al. Methylenetetrahydrofolate reductase genetic polymorphisms in patients with cataract. Am J Ophthalmol. 2005;140(5):932-4.

7. Wang X-b, Qiao C, Wei L, Han Y-d, Cui N-h, Huang Z-l, et al. Associations of Polymorphisms in MTHFR Gene with the Risk of Age-Related Cataract in Chinese Han Population: A Genotype-Phenotype Analysis. PloS one. 2015;10(12).

8. Tan AG, Kifley A, Mitchell P, Rochtchina E, Flood VM, Cumming RG, et al. Associations Between Methylenetetrahydrofolate Reductase Polymorphisms, Serum Homocysteine Levels, and Incident Cortical Cataract. JAMA ophthalmology. 2016.
