# Supplementary material for: Association of OGG1 and MTHFR polymorphisms with age-related cataract: A systematic review and meta-analysis
Source: PLoS One. 2017 Mar 2;12(3):e0172092. doi: 10.1371/journal.pone.0172092 (PMC5333819; doi:10.1371/journal.pone.0172092)
Supplement: S2 Checklist — (DOCX) [file pone.0172092.s007.docx]

**Meta-analysis on Genetic Association Studies Checklist | PLOS ONE**

|  | Item | Section name and paragraph number within manuscript |
| --- | --- | --- |
|  | **Introduction** |  |
| 1 | Provide a detailed justification for the polymorphism studied; if a single polymorphism was analyzed, give details as to why others were not included in the meta-analysis. | Introduction: 3;  Materials and methods-Eligibility criteria:1;  Results-Inclusion of studies for meta-analysis: 1. |
| 2 | Provide a detailed justification for the population(s) and clinical condition studied. | Introduction:1,2,3. |
|  | **Methods** |  |
| 3 | Provide full details of the search strategy employed; outline the full electronic search strategy –specific combination of keywords and any limits applied- for at least one database. Specify whether synonyms of polymorphisms/genes (e.g. SNP number) were searched. | Materials and methods-Literature search: 1. |
| 4 | Report full details on the inclusion and exclusion criteria applied for selecting studies.  *Please list the excluded articles and the reasons for exclusion of each article in a supplementary file.* | Materials and methods-Eligibility criteria: 1, 2.  Excluded articles and reasons are listed in S2 Appendix. |
| 5 | Provide details on how the quality of the studies included in the analyses was assessed. | Materials and methods-Study selection, data collection and risk of bias assessment: 2. |
| 6 | Describe steps taken to contact study authors to identify additional studies and to request missing data. | We didn't contact study authors. |
| 7 | Describe how environmental effects were adjusted for, if this adjustment was not conducted, outline the reasons for this. | Discussion-Study strengths and limitations: 3. |
| 8 | Describe the methods of handling heterogeneity/between-study variance. | Materials and methods-Data analysis:2.  We use subgroup analysis in Results section. |
| 9 | Describe how the Hardy-Weinberg equilibrium and linkage disequilibrium were assessed. | Materials and methods-Study selection, data collection and risk of bias assessment: 1. |
| 10 | Describe and justify the choice of model for the analyses (per-allele vs per-genotype vs genetic model-free, random effects vs fixed effects). | Materials and methods-Data analysis: 1, 2. |
| 11 | Describe whether a sensitivity analysis has been completed. | Materials and methods-Data analysis: 2. |
| 12 | Describe whether an assessment of the effects of population stratification has been conducted. | None. |
| 13 | Describe whether study-specific results have been assessed and if so the reasons for this (e.g. forest plot). | Fig2 |
|  | **Results** |  |
| 14 | Include flow diagram for the studies included in the meta-analysis as the first figure for the manuscript | Results-Inclusion of studies for meta-analysis: 2. |
| 15 | Report details on allele/genotype prevalence. | S3 Table. |
| 16 | Report the effect size estimates and p values for each analysis. | Results-Genetic associations of OGG1 gene with ARC: 1;  Reslts-Genetic association of the MTHFR gene with ARC:1 |
|  | **Discussion** |  |
| 17 | Discuss the limitations of the meta-analysis, including genotyping errors/bias and publication bias. | Discussion-Study strengths and limitations |
| 18 | If the meta-analysis identifies an association within a subgroup of the population studied but not another, discuss the implications of these results, and if applicable the possibility of subgroup-specific publication bias. | None. |
| 19 | Discuss the suitability of the sample size employed to the research question and the power of the study. | Discussion-Meta-analyzed gene and loci |
